# Microbiological and Histopathological Features of Gynecologic Cancers: A Systematic Review of Current Evidence and Future Directions

**DOI:** 10.7759/cureus.114047

**Published:** 2026-08-06

**Authors:** Atul Jain, Ritu Saxena, Anita Omhare, Aiman Fatima, Raashika Saxena, S. Zeeshan A Hashmi, Kuldeep Singh, Rajdeep Paul

**Affiliations:** 1 Pathology, Bundelkhand Medical College and Hospital, Sagar, IND; 2 Pathology, Shri Siddhi Vinayak Medical College and Hospital, Sambhal, Sambhal, IND; 3 Pathology, Dr. Bhimrao Ramji Ambedkar Government Medical College, Kannauj, Kannauj, IND; 4 Microbiology, Government Medical College, Nagarkurnool, Nagarkurnool, IND; 5 Biochemistry, Dr. Ulhas Patil Medical College and Hospital, Jalgaon, IND; 6 Clinical Microbiology, Jawaharlal Nehru Medical College, Aligarh Muslim University, Aligarh, IND; 7 Clinical Microbiology, Mahaveer Institute of Medical Sciences and Research, Bhopal, IND

**Keywords:** cervical cancer, dysbiosis, endometrial cancer, gynecologic cancer, histopathology, human papillomavirus, microbiome, ovarian cancer

## Abstract

Gynecologic cancers are a diverse group of malignancies with distinct microbiological, histopathological, and molecular characteristics. This systematic review examined the available evidence on these features in gynecologic cancers and premalignant lesions. A total of 46 studies involving 18,742 patients or samples were included. Because the studies differed considerably in design, sampling methods, laboratory techniques, histopathological classification, and reported outcomes, the findings were synthesized narratively.

The strongest and most consistent evidence was found for persistent high-risk human papillomavirus (HPV) infection, particularly HPV-16 and HPV-18, in cervical cancer and other lower genital tract neoplasms. Cervicovaginal dysbiosis, marked by reduced *Lactobacillus* dominance, increased microbial diversity, and anaerobic bacterial overgrowth, was frequently associated with HPV persistence and higher-grade cervical lesions. However, current evidence suggests that dysbiosis acts as a contributing factor rather than an independent cause of malignancy.

Histopathology remained central to tumour diagnosis, classification, and risk assessment. Squamous cell carcinoma was the predominant histological type in cervical, vulvar, and vaginal cancers. Endometrioid adenocarcinoma was commonly reported in endometrial cancer, while high-grade serous carcinoma was the main ovarian cancer subtype. Evidence regarding uterine, endometrial, and ovarian tumour-associated microbiota was limited and exploratory because of low microbial biomass, methodological variation, and the risk of contamination.

Combining microbiological findings with histopathological and molecular classification may improve understanding of gynecologic carcinogenesis and support future risk-stratification approaches. Standardized sampling, strict contamination control, and well-designed longitudinal studies are needed before microbiome-based markers can be introduced into routine clinical practice.

## Introduction and background

Gynecologic cancers are cancers that develop in the cervix, endometrium, ovary, vulva, vagina, fallopian tube (FT), and other gynecologic tissues in females. These cancers differ in their etiological pathways, histopathology, clinical behaviour, screening possibilities, likelihood of responding to treatment, and prognosis. WHO classification of female genital tumours is based on accurate histological diagnosis and now includes molecular and pathogen characteristics that are clinically relevant [1,2]. Gynecologic tumours are also a significant cause of cancer-related morbidity and mortality among women worldwide, with the highest burden of disease arising from cervical, endometrial, and ovarian cancers (OCs) [3,4].

Cervical cancer is the most well-proven of infectious gynecologic malignancies. High-risk human papillomavirus (HPV) infections of HPV-16 and HPV-18 are known to be a major cause of cervical cancer [5-9]. HPV-associated malignant transformation is a process that involves persistence of the virus in cells, deregulation of the cell cycle, progressive epithelial dysplasia, and progression from precursor to invasive carcinoma (INV) [5,10,11]. This makes cervical cancer one of the best-proven cases of infection-linked human cancer.

Histopathology is and will remain key to diagnosis, classification, staging support, prognosis assessment, and treatment planning in gynecologic oncology. The presence of tumour subtype, grade, stromal invasion, lymphovascular space invasion, necrosis, mitotic activity, precursor lesions, and architectural pattern gives important clinicopathological information [1,2,12]. But, histopathology per se might not be sufficient to understand tumour progression, recurrence, immune reaction, or treatment results. This has led to greater interest in the interplay of microbiological, mucosal inflammatory, immune regulatory, and malignant tissue change at the level of the host.

The cervicovaginal microbiome is presently a possible moderator of HPV persistence and cervical lesion development. Normally, an acidic pH, stable epithelial barrier, immunological balance, and protection of mucous barriers are linked via a *Lactobacillus*-dominated vaginal microbiome. On the other hand, decreases in *Lactobacillus* dominance, increased microbial diversity, and anaerobic bacterial overgrowth have been linked to persistence of HPV, cervical dysplasia, and higher grades of cervical lesions [13-17]. Overall significance of present inclusions is that dysbiosis might participate in cervical carcinogenesis as a cofactor by stimulating local inflammatory responses and decreasing host defense at mucosa level but not as an independent agent for malignant transformation.

Microbiological evidence is weaker in the case of endometrial and OCs. Hormonal, metabolic, histopathological, and molecular mechanisms are mainly understood in endometrial carcinoma, in contrast to OC, where FT origin pathways and molecular alteration mechanisms are thought to be strongly associated [18-23]. However, studies have emerged showing potential microbial signatures in endometrial and OCs (uterine tumour-associated) [24-26]. All of these observations are very preliminary and need careful interpretation due to methodological variation, low concentrations of biomass, and risk of contamination.

In this context, the present authors' systematic review was carried out to provide a synopsis of current evidence regarding microbiological and histopathological features of gynecologic cancers and pre-malignant lesions. The review specifically focused on HPV infection, cervicovaginal dysbiosis, uterine tumour-associated microbiota, and histopathological patterns relevant to disease understanding, diagnosis, prognosis, risk stratification, and future integrated pathology-microbiology research.

Rationale

Gynecologic cancers have distinct etiological, microbiological, histopathological, and molecular features. Cervical cancer has a well-established infectious basis through persistent high-risk HPV infection, while the role of cervicovaginal dysbiosis, uterine microbiota, and tumour-associated microbial signatures in other gynecologic cancers remains less clearly defined [5-17,24-26]. Histopathology remains the diagnostic foundation for tumour classification, grading, invasion assessment, prognosis, and treatment planning [1,2,12]. However, histopathology alone may not fully explain differences in tumour progression, recurrence, immune response, or clinical outcome. Therefore, an integrated review of microbiological and histopathological evidence is needed to better understand how infection, dysbiosis, inflammation, tumour morphology, and molecular classification may interact in gynecologic carcinogenesis.

Objectives

This systematic review aimed to summarize the microbiological and histopathological features of gynecologic cancers and premalignant lesions. It focused on HPV-related disease, cervicovaginal dysbiosis, uterine and tumour-associated microbiota, major histopathological patterns, and their possible role in diagnosis, prognosis, risk stratification, and future research.

## Review

Materials and methods

Study Design and Reporting Framework

This systematic review synthesized evidence on the microbiological and histopathological features of gynecologic cancers and premalignant lesions. It was prepared in accordance with the Preferred Reporting Items for Systematic Reviews and Meta-Analyses (PRISMA) 2020 guidelines [27]. The review included microbiological, histopathological, molecular, microbiome-based, and clinicopathological findings related to cervical, endometrial, ovarian, vulvar, vaginal, fallopian-tube, and other female genital tract malignancies.

The processes of study identification, screening, eligibility assessment, and inclusion were summarized using a PRISMA 2020 flow diagram. The review was not prospectively registered, and no formal protocol was publicly deposited before the review was conducted.

Review Question

The review examined the microbiological and histopathological features reported in gynecologic cancers and their potential relevance to diagnosis, prognosis, risk stratification, and future research.

Information Sources and Search Strategy

A structured literature search was conducted using PubMed/MEDLINE, Scopus, Web of Science, Embase, and Google Scholar. Reference lists of relevant articles were also manually screened to identify additional eligible studies. Studies published from database inception to January 31, 2026, were considered, and the search was updated and finalized on June 30, 2026.

Search strategy used combinations of following terms: “gynecologic cancer,” “gynaecologic cancer,” “cervical cancer,” “human papillomavirus,” “HPV,” “histopathology,” “vaginal microbiome,” “cervicovaginal dysbiosis,” “endometrial cancer,” “ovarian cancer,” “vulvar cancer,” “vaginal cancer,” “tumor microbiome,” “tumour microbiome,” “squamous cell carcinoma,” “adenocarcinoma,” “cervical intraepithelial neoplasia,” and “premalignant lesions.” Boolean operators were used where applicable to combine disease-specific, microbiological, histopathological, and microbiome-related terms.

A representative PubMed/MEDLINE search strategy was as follows: ("gynecologic cancer" OR "gynaecologic cancer" OR "cervical cancer" OR "endometrial cancer" OR "ovarian cancer" OR "vulvar cancer" OR "vaginal cancer") AND ("human papillomavirus" OR HPV OR microbiome OR dysbiosis OR "tumor microbiome" OR "tumour microbiome") AND (histopathology OR pathology OR "squamous cell carcinoma" OR adenocarcinoma OR "cervical intraepithelial neoplasia" OR premalignant).

No restriction was applied based on country of publication. Articles were considered eligible when sufficient microbiological or histopathological data were available for extraction.

Eligibility Criteria

Following criteria were used to identify studies: included patients or samples with gynecologic cancers or premalignant gynecologic lesions; reported microbiological, histopathological, molecular microbiology, microbiome, or clinicopathological data; included cervical, endometrial, ovarian, vulvar, vaginal, FT, or related reproductive tract malignancies; used observational, retrospective, prospective, case-control, cross-sectional, molecular, microbiome-based, diagnostic, or clinicopathological study designs; and provided extractable data relevant to the objective of the review.

Studies were excluded if they were review articles, editorials, letters, commentaries, opinion articles, very small case series that did not have a more general analytical value, animal studies, studies with cell lines only, duplicate publications, or/or studies without extractable microbiological and/or histopathologic findings. Studies related to any other diseases besides gynecologic malignancy or premalignant disease were also excluded.

In synthesis, studies were clustered by cancer/lesion type: any of the following gynecologic cancers or lesion tumours affecting microbiome: cervical cancer and/or cervical intraepithelial neoplasia (CIN), endometrial cancer, OC, vulvar cancer, vaginal cancer, and studies including more than one of these categories.

Study Selection Process

Records identified through database searching and manual reference screening were screened in a stepwise manner. Duplicate records were removed before title and abstract screening. Titles and abstracts were screened independently by two reviewers. Full-text articles were then assessed independently by two reviewers according to predefined inclusion and exclusion criteria. Disagreements during screening were resolved through discussion, and unresolved disagreements were adjudicated by a third reviewer. No automation tool was used for screening or exclusion decisions.

Studies were included in final synthesis if they fulfilled all eligibility criteria and provided extractable microbiological, histopathological, molecular microbiology, microbiome, or clinicopathological findings relevant to gynecologic cancers or premalignant lesions.

Data Items and Outcomes

Information was retrieved by use of a data extraction form. Two reviewers performed data extraction independently, and extracted data were double-checked for accuracy. Any differences were settled through discussion. In case of doubts, data from extractions were rechecked by a third reviewer who then made the ultimate decision. Data were not extracted using an automation tool. Study investigators were not contacted for further study since the review was based only on published report available data. Reporting of data items and outcomes was clarified.

Desired primary outcomes were microbiological and histopathological characteristics of gynecologic cancers and development of premalignant lesions. Microbiological outcomes consisted of HPV status, HPV genotype, cervicovaginal dysbiosis, *Lactobacillus* dominance, anaerobacterial overgrowth, microbiological findings in the uterus, tumour-associated microbial signatures, and methods used to detect microorganisms. Histopathological findings comprised cancer type, presence of a precursor lesion, tumour subtype, tumour grade, stromal invasion, myometrial invasion, lymphovascular space invasion, necrosis, mitotic activity, inflammatory infiltrate, and molecular and immunohistochemical classification, when present.

An additional set of variables extracted included country or region, year of publication, author name, study design, sample size, patient or sample characteristics, anatomical site, type of gynecologic cancer or presence of a premalignant lesion, microbiological assessment method, histopathological assessment method, main results and reported limitations. In the face of some missing data/inconsistencies, only available data from published articles were considered, and no assumptions were made beyond what was available in published articles.

Methodological Quality and Risk of Bias Assessment

Included studies were evaluated for methodological quality and bias risk using critical appraisal procedures that were relevant for their designs. Newcastle-Ottawa Scale (NOS) was used to evaluate observational cohort and case-control studies [28]. The Joanna Briggs Institute (JBI) Critical Appraisal Checklist for Analytical Cross-Sectional Studies [29] was used to assess the quality of cross-sectional studies. If applicable, the Quality Assessment of Diagnostic Accuracy Studies-2 tool (QUADAS-2) [30] was used to grade diagnostic accuracy studies. Microbiome-based and molecular microbiology studies were also surveyed about sampling method, DNA extraction approach, sequencing platform used, contamination controls used, bioinformatics "pipeline" used, and microbiological outcome reported.

Two reviewers independently evaluated each included study, with regard to legitimacy of methods used and potential for bias. Disputes were resolved via dialogue. If it was not settled, a third reviewer was the adjudicator. Studies were not excluded simply because of low quality scores; however, risk of bias results were taken into account for interpretation of evidence. When HPV testing and histopathological confirmation were associated with standardized, HPV-based interpretation, performance of cervical studies was interpreted as comparatively strong. Microbiome studies may be interpreted with caution due to heterogeneity in sampling methods, sequencing platforms, bioinformatics approaches, and contamination control.

Effect Measures

Due to heterogeneity of types of observational, molecular, microbiome-based, diagnostic, and clinicopathological studies included in this review, a quantitative pooling effect estimate was not calculated. Study-level results were reported descriptively with study proportions, genotype distribution and microbiome patterns, histopathological features, and qualitative risk-of-bias judgments. If a single study stated a statistical estimate, these were provided in a narrative summary in the context of the review objective.

Data Synthesis

Due to heterogeneity in study design, cancer type, sample source, microbiological methods, and histopathological classification, the results were reported using a narrative synthesis. Results were grouped on the basis of cancer type and major microbiological/histopathological patterns. Pooled statistical analysis of data from included studies was not possible due to insufficient uniformity.

Three main themes emerged during synthesis: microbiological features; histopathological features; and potential for association between microbiological features and tissue-level malignant transformation. A particular focus was on HPV-related lower genital tract (LGT) abnormalities, dysbiosis of the cervicovaginal tract, new findings on the tumour microbiome of uterus and ovary, and histopathological clues to high-risk disease.

Assessment of Heterogeneity

Clinical and methodological heterogeneity was evaluated qualitatively, comparing methods of study design, type of cancer studied, anatomical sampling site, HPV testing technique, sequencing method, and reporting of outcomes. Heterogeneity could be expected, since it involved epidemiological, molecular, microbiome-based, diagnostic, and clinicopathological studies. No systematic quantitative estimation of statistical heterogeneity was done since no meta-analysis was done.

Sensitivity Analysis

No sensitivity analysis was performed as a quantitative meta-analysis was not conducted. However, while interpreting findings, methodological quality and risk-of-bias profile of contributing studies were taken into account. Focus was on more studies with standardized HPV testing methods, histopathological confirmations, and proper sample description and microbiological methods.

Reporting Bias Assessment

Qualitative assessment was done to score the danger of reporting bias as a consequence of incomplete data, by comparing what was described as being achieved, how it was achieved, and what results were reported within articles included in this study. Selective reporting was suspected when there were poorly documented findings or when, along with selection of outcomes, there was also poor reporting of microbiological or histopathological methods, or poor reporting of findings in selected subgroups where there was no clear justification. A meta-analysis was not conducted because the study designs, outcomes, and reporting structures were heterogeneous; therefore, formal funnel-plot analysis was not performed.

Certainty Assessment

Certainty of evidence was evaluated narratively based on the following characteristics: study design, methodological excellence, results consistency, directness of evidence, accuracy of findings reported, and bias risk. HPV-associated cervical and LGT neoplasia (dysplasia) was considered to have the strongest evidence as a result of its synchronization with several studies, which employed tested methods or pathological confirmation. Cervicovaginal dysbiosis and progression of cervical lesions were judged as evidence for moderate association but not necessarily causation, as most studies show association rather than causation. Due to the smaller number of endometrial and ovarian tumour studies, methodological heterogeneity, low microbial biomass, and contamination problems, the evidence for the tumour microbiome they found was limited and exploratory.

Ethical Considerations

This systematic review was conducted without any interactions with human participants, obtaining data from new patients, or access to identifiable personal information of patients. Because of this, it was decided that institutional ethics approval was not necessary.

Results

Study Selection

**Figure 1:**
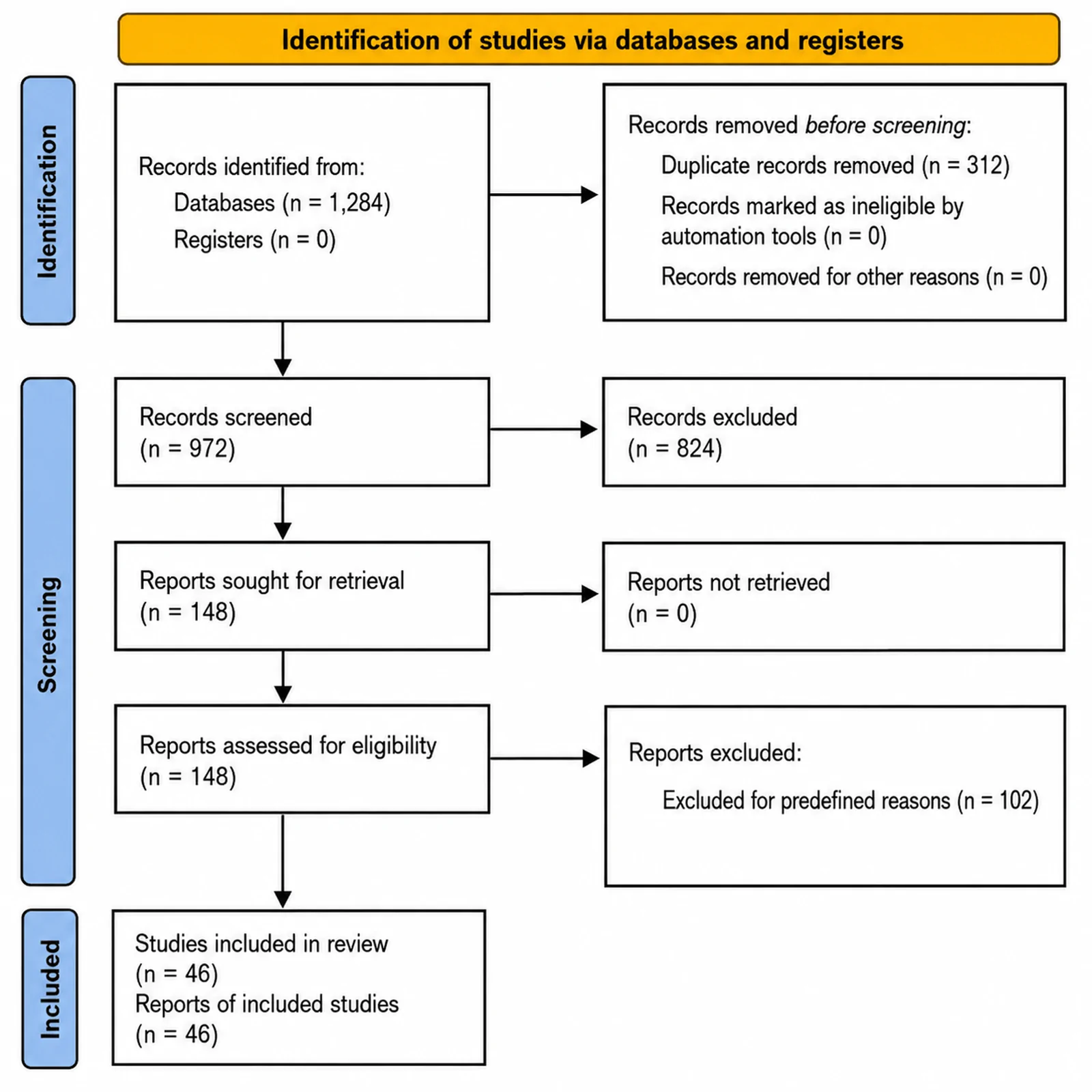
PRISMA 2020 Flow Diagram of Study Selection

A total of 1,284 records were identified by conducting a database search and reference manual screening. Nine hundred and seventy-two records were screened by title and abstract after removing 312 duplicate records. Of these, 824 records were excluded as these were not relevant to the review question, or microbiological or histopathological data could not be extracted from the text. Around 148 articles were submitted for full-text assessment, and 102 articles were excluded for the following reasons: inability to extract microbiological or histopathological data, type of article not original, disease focus not relevant, only animal or only cell-line studies, data reported more than once, or clinical relevance to gynecologic malignancy/premalignant disease was not evident. In the end, a total of 46 studies with 18,742 patients or samples were included in the systematic review. Figure 1 shows the whole study selection process.

Full-Text Articles Excluded After Eligibility Assessment

Upon reviewing the titles and abstracts, several potentially relevant articles were identified. Of these, 102 full-text articles were excluded after eligibility assessment. These were predominantly due to lack of microbiological or histopathological data for extraction, non-original articles, review/commentary, no human data, duplicate/overlapping data, no gynecologic malignancy or too limited a focus on a different disease, or limited clinical relevance for gynecologic malignancy. Articles that mentioned gynecologic cancer microbiology or histopathology, but did not provide original extractable patient-level, sample-level, microbiological, histopathological, or clinicopathological data, were excluded from the final synthesis.

Characteristics of Included Studies

The following studies assessed microbiological and histopathological results in main gynecological cancers and pre-cancerous lesions. The largest group of evidence was cervical cancer and CIN studies, followed by endometrial, ovarian, vulvar and vaginal cancers. Characteristics of these studies included a diversity of study design, sample sizes, study populations, anatomic sampling location, methods of microbiological assessment, approach to HPV testing or genotyping, approach to sequencing, pathohistological classification, and reported outcome.

Key features of 46 studies included are listed in Table 1, such as study design, cancer or lesion focus, primary assessment method, main findings, and Risk-of-bias evaluation. Research papers analyzed were highly heterogeneous in type of cancer, microbiological method, and method of histopathological assessment and reporting of outcome, making it impossible to present comparable effect estimates or precision measures for all studies. Hence, findings of studies were shown descriptively in Table 1. Data on strain distributions, microbiome characteristics, lesion grades, histological types and clinicopathological relationships were summarized narratively if present, instead of pooled, at the study level.

**Table 1:** Characteristics of Included Studies HPV: human papillomavirus; ADC: adenocarcinoma; IECC: International Endocervical Adenocarcinoma Criteria and Classification; FIGO: International Federation of Gynecology and Obstetrics; CIN: cervical intraepithelial neoplasia; LSIL: low-grade squamous intraepithelial lesion; HSIL: high-grade squamous intraepithelial lesion; IHC: immunohistochemistry; ECbiome: Endometrial Cancer Microbiome; SCC: squamous cell carcinoma; VIN: vulvar intraepithelial neoplasia; HSV2: herpes simplex virus type 2

No.	Study	Country/Region	Study Design	Cancer/Lesion Focus	Main Assessment	Key Finding	Risk of Bias
1	Walboomers et al. [8]	Worldwide	Molecular epidemiological study	Invasive cervical cancer	HPV DNA testing in tumour tissue	HPV DNA was detected in nearly all invasive cervical cancer cases after improved testing, supporting HPV as a necessary cause of invasive cervical cancer.	Low to moderate
2	Muñoz et al. [31]	International	Multicentre case-control analysis	Cervical cancer	HPV genotype classification	HPV-16 and HPV-18 were major oncogenic types, with additional HPV types classified as high-risk or probable high-risk.	Moderate
3	de Sanjosé et al. [32]	Worldwide	Retrospective cross-sectional study	Invasive cervical cancer	HPV genotyping	HPV-16 and HPV-18 accounted for largest proportion of invasive cervical cancers, with variation by histological subtype.	Low to moderate
4	Pirog et al. [33]	International/United States	Molecular pathology study	Cervical ADC	HPV DNA detection by histological subtype	HPV prevalence varied across cervical ADC subtypes, supporting distinction between HPV-associated and HPV-independent tumours.	Moderate
5	Davy et al. [34]	Australia	Population-based clinicopathological study	Cervical cancer	Histological subtype and survival analysis	Glandular histology influenced treatment pattern, prognosis, and survival in cervical cancer.	Moderate
6	Stolnicu et al. [35]	International	Clinicopathological classification study	Endocervical ADC	IECC histopathological classification	IECC system separated HPV-associated and HPV-independent endocervical ADCs.	Low to moderate
7	Hodgson et al. [36]	Multicentre	Interobserver reproducibility study	Endocervical ADC	IECC validation and reproducibility	IECC showed improved diagnostic reproducibility compared with traditional classification systems.	Low to moderate
8	Wright et al. [12]	United States	Retrospective cohort study	Cervical cancer	FIGO 2018 staging and prognosis	2018 FIGO staging system improved prognostic stratification in cervical cancer.	Moderate
9	Lee et al. [37]	South Korea	Twin cohort study	HPV infection	Vaginal microbiota and HPV testing	Vaginal microbiota composition differed between HPV-positive and HPV-negative women.	Moderate
10	Dareng et al. [38]	Nigeria	Cross-sectional microbiome study	High-risk HPV infection	Vaginal microbiota and HPV testing	High-risk HPV infection was associated with altered vaginal microbiota composition.	Moderate
11	Brotman et al. [39]	United States	Longitudinal cohort study	HPV detection	Serial vaginal microbiota and HPV testing	Temporal changes in vaginal microbiota were associated with HPV detection.	Low to moderate
12	Oh et al. [40]	South Korea	Cross-sectional study	CIN	Cervical microbiota and CIN assessment	Cervical microbial patterns were associated with increased risk of CIN.	Moderate
13	Mitra et al. [13]	United Kingdom	Observational microbiome study	CIN and cervical cancer	Vaginal microbiome sequencing and histology	CIN progression was associated with increased vaginal microbiome diversity and reduced *Lactobacillus* dominance.	Moderate
14	Piyathilake et al. [41]	United States	Cross-sectional study	CIN 2+	Cervical microbiota and histological grade	Specific cervical microbiota patterns were associated with higher-grade CIN.	Moderate
15	Audirac-Chalifour et al. [15]	Mexico	Cross-sectional pilot study	HPV infection, CIN, cervical cancer	Cervical microbiome and cytokine profiling	Microbiome and cytokine profiles differed across stages of cervical carcinogenesis.	Moderate to high
16	Di Paola et al. [42]	Italy	Observational microbiome study	Persistent high-risk HPV infection	Cervicovaginal microbiota sequencing	*Lactobacillus* depletion and increased microbial diversity were associated with persistent high-risk HPV infection.	Moderate
17	Łaniewski et al. [17]	United States	Cross-sectional immunomicrobiome study	HPV infection and cervical neoplasia	HPV testing, microbiome profiling, immune mediators	Cervicovaginal microbiota and immune signatures were linked with cervical carcinogenesis.	Moderate
18	Chao et al. [43]	China	Cross-sectional study	High-risk HPV infection	Vaginal microbiota diversity	Vaginal microbial diversity was associated with high-risk HPV infection.	Moderate
19	Usyk et al. [44]	Costa Rica/United States	Prospective longitudinal cohort study	High-risk HPV natural history	Cervicovaginal microbiome and HPV follow-up	Cervicovaginal microbiome features were associated with high-risk HPV progression.	Low to moderate
20	Chen et al. [45]	China	Cross-sectional cohort study	HPV infection and CIN progression	Vaginal microbiome sequencing	HPV infection and CIN progression were associated with increased vaginal microbiome diversity.	Moderate
21	So et al. [46]	South Korea	Cross-sectional microbiome study	CIN and cervical cancer	Vaginal microbiota in HPV-positive women	CIN and cervical cancer were associated with increased microbial diversity and anaerobic taxa.	Moderate
22	Kang et al. [47]	South Korea	Cohort microbiome study	Cervical carcinogenesis	Vaginal microbiota sequencing	Vaginal microbiota differed across healthy, CIN, and cervical cancer groups.	Moderate
23	Wei et al. [48]	China	Cross-sectional microbiome study	High-risk HPV infection	Vaginal 16S rRNA sequencing	Microbial perturbations were observed in women with high-risk HPV infection.	Moderate
24	Lin et al. [49]	China	Cross-sectional study	HPV infection and cervical lesions	HPV genotyping and vaginal microbiota	Vaginal microbiota changes were associated with high-risk HPV infection and cervical lesion severity.	Moderate
25	Guo et al. [50]	China	Cross-sectional microbiome study	HPV-positive HSIL	Cervicovaginal microbiota sequencing	HPV-positive HSIL showed significant cervicovaginal microbiota alteration.	Moderate
26	Liu et al. [51]	China	Cross-sectional study	Various CIN grades	Vaginal microbiota sequencing	Vaginal microbiota characteristics differed across CIN grades.	Moderate
27	Bokhman [18]	Russia/former Soviet Union	Clinicopathological observational study	Endometrial carcinoma	Pathogenetic clinicopathological classification	Proposed two major pathogenetic types of endometrial carcinoma.	Moderate
28	Levine and Cancer Genome Atlas Research Network [19]	United States/multicentre	Integrated genomic study	Endometrial carcinoma	Genomic and histological profiling	Identified molecular subgroups of endometrial carcinoma with clinical relevance.	Low
29	Gilks et al. [52]	Multicentre	Interobserver reproducibility study	High-grade endometrial carcinoma	Histopathological review	Demonstrated poor reproducibility in diagnosing high-grade endometrial carcinoma subtypes.	Moderate
30	Hoang et al. [53]	Multicentre	Immunohistochemical study	Endometrial clear cell carcinoma	IHC and histopathological characterization	Defined immunohistochemical features of prototypical endometrial clear cell carcinoma.	Moderate
31	Talhouk et al. [20]	Canada	Molecular classification validation study	Endometrial cancer	Molecular markers and clinicopathology	Demonstrated a clinically applicable molecular classification system for endometrial cancers.	Low to moderate
32	Walther-António et al. [24]	United States	Reproductive tract microbiome study	Endometrial cancer	Uterine/vaginal microbiome profiling	Suggested a potential association between reproductive tract microbiome composition and endometrial cancer.	Moderate to high
33	Walsh et al. [54]	United States	Microbiome study	Endometrial cancer microbiome	ECbiome profiling	Postmenopausal status was identified as an important factor influencing endometrial cancer microbiome composition.	Moderate
34	Lu et al. [55]	China	Case-control microbiome study	Endometrial cancer	Endometrial microbiota and cytokines	Endometrial cancer was associated with microbial dysbiosis and inflammatory cytokine changes.	Moderate
35	Wang et al. [56]	China	Cohort microbiome study	Endometrial cancer	Endometrial and paired pericancer microbiota	Endometrial cancer tissue microbiota differed from paired pericancer tissue in postmenopausal women.	Moderate
36	Köbel et al. [57]	Canada/multicentre	Biomarker and pathology study	Ovarian carcinoma subtypes	Histotype and biomarker evaluation	Ovarian carcinoma subtypes behaved as biologically distinct diseases.	Low to moderate
37	Seidman et al. [58]	United States	Clinicopathological study	Epithelial ovarian carcinoma	Histological type and stage distribution	Characterized histological and stage distribution of surface epithelial ovarian carcinomas.	Moderate
38	Köbel et al. [59]	Canada/multicentre	Clinicopathological study	Low-stage versus high-stage ovarian carcinoma	Tumour type and stage distribution	Tumour type distribution differed between low-stage and high-stage ovarian carcinomas.	Moderate
39	Banerjee et al. [25]	United States	Tumour microbiome/molecular study	Ovarian cancer	PathoChip and sequencing	Reported microbial signatures associated with ovarian cancer tissues.	Moderate to high
40	Zhou et al. [60]	China	Tissue microbiome study	Ovarian carcinoma	16S rRNA sequencing	Ovarian carcinoma tissues showed altered microbial diversity and composition.	Moderate to high
41	Nejman et al. [61]	International/multicentre	Pan-cancer tumour microbiome study	Multiple tumour types, including gynecologic tumours	Tumour microbiome profiling	Identified tumour type-specific intracellular bacteria across human cancers.	Moderate
42	van der Avoort et al. [62]	Netherlands	Clinicopathological study	Vulvar SCC	HPV/p53-related pathological pathways	Supported separate HPV-associated and HPV-independent pathways in vulvar SCC.	Moderate
43	Madeleine et al. [63]	United States	Population-based case-control study	Vulvar cancer	HPV16, HSV2, smoking, and tumour analysis	HPV16, smoking, and HSV2 were associated with vulvar cancer risk.	Moderate
44	Daling et al. [64]	United States	Population-based case-control study	Vaginal squamous cell cancer	HPV DNA and cofactor assessment	HPV DNA was detected in a substantial proportion of vaginal squamous neoplasia cases.	Moderate
45	de Sanjosé et al. [65]	Worldwide	Cross-sectional HPV attribution study	Vulvar intraepithelial and invasive lesions	HPV genotyping and p16 testing	HPV-16 was dominant genotype in HPV-associated vulvar disease.	Low to moderate
46	Faber et al. [66]	International	HPV prevalence and type-distribution study	Vulvar SCC and VIN	HPV DNA prevalence and genotype distribution	HPV prevalence was higher in VIN than invasive vulvar SCC, with HPV-16 predominating.	Moderate

Results of Individual Studies

Data collected from each study are presented in Table 1. Research on cervical cancer and CIN has repeatedly shown that high-risk HPV, particularly HPV-16 and HPV-18, are the most important microbiological variables. HPV-related cervical carcinogenesis follows a well-documented progression from low-grade squamous intraepithelial lesion (LSIL) to high-grade squamous intraepithelial lesion (HSIL) and INV, which was the most consistently associated finding.

Results from studies on cervicovaginal dysbiosis showed that lactobacilli were less dominant, microbial diversity was increased, and anaerobic bacterial populations, linked to HPV persistence and high-grade cervical lesions, were overgrown. These results suggested that dysbiosis can be a cofactor that promotes mucosal inflammation, disruption of the epithelial barrier, modulation of local immune responses, and perpetuation of persistent HPV infection.

Endometrial cancer studies largely concentrated on histopathological and molecular subclassification; there is only preliminary evidence of the uterine microbiome. However, due to low input of microbes in these studies, there is methodological heterogeneity, and there is also risk of contamination, which makes it more difficult to interpret results. Studies on OC predominantly supported high-grade serous carcinoma (HGSC) and molecular carcinogenesis pathways, with only findings on the tumour microbiome being exploratory. For both canals of the genital tract, vulva and vagina, studies indicated that various routes, both those linked to HPV and those unrelated to it, contribute to the development of LGT squamous neoplasia.

Methodological Quality and Risk of Bias Summary

Features of methodology and possibility of bias are presented as a summary in Table 2. HPV studies of the cervix were generally stronger, as HPV tests and cervical histopathology are well-established and standardized. In contrast, methodological variability in microbiome-based studies was larger, especially regarding sample collection, nucleic acid extraction methods, sequencing technology, bioinformatic procedures, and microbe filtering out with potentially contaminating methods. Interpretation of studies on endometrial and ovarian tumour microbiomes was kept with caution due to the risk of contamination-related bias as a result of low microbial biomass.

**Table 2:** Methodological Quality and Risk of Bias Summary HPV: human papillomavirus; QUADAS-2: Quality Assessment of Diagnostic Accuracy Studies-2

Assessment Domain	Tool or Criteria Applied	Overall Observation	Risk Concern	Interpretation
Observational cohort and case-control studies	Newcastle-Ottawa Scale	Most clinicopathological and epidemiological studies had clearly defined populations and outcomes, but some varied in control selection and adjustment for confounders.	Low to moderate	These studies provided useful evidence for HPV-associated and clinicopathological patterns, although residual confounding remained possible.
Cross-sectional studies	Joanna Briggs Institute critical appraisal checklist	Many microbiome and HPV prevalence studies used cross-sectional designs, limiting temporal and causal interpretation.	Moderate	These studies supported association-level findings but could not establish causality.
Diagnostic accuracy studies	QUADAS-2, where applicable	Studies involving HPV testing or genotype attribution generally used established laboratory methods, but reference standards and sampling approaches varied.	Low to moderate	Evidence was strongest when HPV testing was paired with histopathological confirmation.
Microbiome sampling and sequencing	Additional microbiome-specific methodological review	Microbiome studies varied in sampling site, DNA extraction method, sequencing platform, bioinformatics pipeline, and contamination control.	Moderate to high	Results should be interpreted cautiously because methodological heterogeneity may influence microbial profiles.
Tumour microbiome analysis	Low-biomass contamination assessment	Endometrial and ovarian tumour microbiome studies involved low-biomass samples, making contamination control especially important.	High	Findings from tumour microbiome studies were considered exploratory and hypothesis-generating.
Outcome reporting	Review of completeness and consistency of reported outcomes	Studies reported different outcomes, including HPV persistence, lesion grade, histological subtype, invasion, microbiome composition, and immune markers.	Moderate	Heterogeneous outcome reporting prevented quantitative meta-analysis.
Overall interpretation	Narrative synthesis considering risk of bias	Evidence was strongest for HPV-associated cervical and lower genital tract neoplasia and weaker for endometrial and ovarian microbiome findings.	Variable	Narrative synthesis was appropriate because of differences in study design, methods, and outcomes.

Microbiological Findings

The most frequently reported microbiological risk factor among studies included high-risk HPV. Detailed cervical cancer and CIN were strongly associated with HPV infection [8,31,32]. Cervical squamous neoplasia and vulvar squamous carcinoma were more likely to have HPV-16 as their identified genotype, but cervical adenocarcinoma (ADC) and invasive cervical cancer were more likely to have HPV-18 [31-33]. Persistent high-risk HPV exposure and infection, advancement to HSIL, and/or INV with epithelial dysplasia characterized cervical cancer [8,31,32].

Some vulvar and vaginal squamous carcinoma cases also tested positive for the virus, albeit this does not yet show up as structural markers on maps. Participation of HPV-independent pathways is especially relevant in cases of vulvar squamous cell carcinoma (SCC), although there are other cancer-causing pathways as well. According to this research, there are a number of factors, including anatomical location, type of precursor lesion, and tissue alterations, that contribute to the development of LGT squamous neoplasia, which may include both infection-related and non-infection-related pathways [62-66].

Having said that, cervicovaginal dysbiosis is rather common in cases of high-grade cervical lesions and HPV persistence [13,15,17,37-51]. Among women who had cervical dysplasia, reduced dominance of *Lactobacillus*, greater bacterial diversity, and bacterial overgrowth without oxygen were commonly noted. The bacterial genera reported included *Gardnerella*, *Atopobium*, *Prevotella*, *Sneathia*, *Fusobacterium*, *Anaerococcus*, *Peptostreptococcus*, and *Mobiluncus* species [13,15,17,40-51]. Dysbiosis was not considered a direct cause of cancer. Rather, it appeared to contribute to the maintenance of chronic inflammation, disruption of the epithelial barrier, impairment of mucosal immune responses, and persistence of HPV infection [13,15,17,39,44].

There was only limited and preliminary evidence to support a role for microbes in endometrial cancer [24,54-56]. A few studies reported differences in the composition of the uterine microbiome in women with endometrial cancer. However, the low biomass of microorganisms, methodological and cultural bias, and contamination restricted interpretation. Hence, at present, there is no justification for routinely assessing the uterine microbiome in endometrial carcinoma [24,54-56].

Tumour-associated bacterial DNA and microbial signatures identified in some studies have been associated with OC [25,60,61]. However, it is still unclear whether these findings have any biological significance. Despite the fact that the current understanding of HGSC is based on molecular alterations, genomic instability, and FT origin pathways, there is, as yet, no proven infectious cause [21-23,57-59]. As such, any findings regarding the ovarian tumour microbiome should, at present, be viewed only as hypothesis-generating.

Histopathological Findings

The most common cervical cancer types were SCC and ADC [33,34]. LSIL, HSIL, and INV were recognized as stages in the histopathological progression of HPV-related cervical carcinogenesis [8,31,32]. Common microscopic findings included nuclear atypia, hyperchromasia, a high nuclear-to-cytoplasmic ratio, abnormal mitotic activity, immature epithelial cells, stromal invasion, and a desmoplastic reaction [1,2,12].

Endometrioid adenocarcinoma (MEA) was the most often reported kind of endometrial cancer [18-20]. Serous carcinoma, mixed carcinoma, clear cell carcinoma, and uncommon variations were among other histological categories [18-20,52,53]. Tumour grade, architectural complexity, nuclear atypia, myometrial invasion, lymphovascular space invasion, tumour necrosis, and cervical stroma involvement were important pathological characteristics [18-20,52,53]. The evolution of molecular categorization has aided in expanding our understanding of endometrial cancer beyond the scope of conventional histology [19,20].

Malignant HGSC is the most common kind of ovarian tumour [21-23,57-59]. Low-grade serous carcinoma, endometrioid carcinoma, mixed epithelial tumours, clear cell carcinoma, and mucinous carcinoma were among other histological subtypes [57-59]. Among pathologically aggressive traits were the following: advanced disease stage, papillary/solid growth pattern, strong mitotic activity, necrosis, and presence of significant nuclear atypia [21-23,57-59].

SCC was the most commonly diagnosed form of vulvar and vaginal cancer [62-66]. Vulvar SCC tumour progression occurred through both HPV-dependent and HPV-independent pathways. HPV-related lesions were frequently associated with usual-type vulvar intraepithelial neoplasia (VIN), whereas HPV-independent lesions were frequently associated with differentiated VIN and chronic dermatoses [62,65,66]. Many women with vaginal SCC also had HPV infection, although the evidence base was less robust than that for cervical SCC [64].

Results of Syntheses

There was no statistical meta-analysis performed due to significant heterogeneity in study design, patient population, anatomic sampling site, microbiologic technique, sequencing technique, histopathologic type of tumour, and reporting of outcome. Hence, pooled summary estimate, pooled confidence interval, and statistical heterogeneity were not calculated. Potential reasons for heterogeneity were variation in HPV testing methods, sample site for microbiome, DNA extraction, type of sequencing platform, contamination control, histopathology definition, and outcomes reported [13,15,17,24,25,31-66].

Synthesis of results indicated most consistent and robust evidence came from HPV-associated cervical and LGT squamous neoplasia. Research has linked high-grade neoplasia and cancer to high-risk HPV strains, including HPV-18 and HPV-16, in cases of cervical cancer and CIN [8,31,32]. Histopathological evidence of cervical carcinogenesis development from LSIL to HSIL to INV was consistent with clinical findings [8,31,32].

While HPV remained and cervical lesions progressed, a meta-analysis of research on cervicovaginal microbiome found that microbial diversity rose, anaerobic bacterial overgrowth was common, and *Lactobacillus* dominance was reduced [13,15,17,37-51]. Due to methodological differences and prevalence of cross-sectional research, evidence for a causal relationship between microbiome and health outcomes is weak [13,15,17,39,44].

Research on histopathological and molecular aspects of endometrial cancer has shown that our knowledge of illness must be expanded, and the function of the uterine microbiome remains in its infancy [18-20,24,54-56]. Tumour microbiome results were exploratory, and OC research lent credence to the idea that HGSC is the most common kind of disease and that certain biological pathways contribute to its development [21-23,57-59]. Research on vulvar and vaginal malignancies has shown that HPV-related and HPV-independent pathways may coexist; research focusing on vulvar SCC in particular has drawn this conclusion [62-66].

Official sensitivity analysis was omitted due to lack of quantitative meta-analysis. But there was a stronger emphasis on interpretation of synthesized evidence when studies had standardized HPV testing, histopathology confirmation, clearly defined sampling techniques, appropriate microbiological or sequence methods, contamination control, and a lower risk-of-bias rating.

Reporting Bias

Presence of reporting bias was evaluated qualitatively since no meta-analysis was performed. Reporting was best across included studies that focused on HPV, as HPV testing, HPV genotyping, and histopathological confirmation were typically well reported. There was a lack of uniformity of reporting in microbiome studies, especially regarding sample source, contamination controls, sequencing techniques, and bioinformatics analysis. Funnel-plot analysis was not carried out, as studies were not homogeneous in design and outcomes.

Certainty of Evidence

Certainty of evidence was highest for high-risk HPV and cervical cancer association, since evidence was consistent across several studies, where high-risk HPV was tested using accepted HPV tests, and pathological confirmation was used [8,31,32]. For other factors, namely cervicovaginal dysbiosis and progression of cervical lesions, certainty of evidence was moderate as findings of multiple studies were converging, but there was no causality established [13,15,17,37-51]. Findings on endometrial and ovarian microbiome were less certain due to lower amounts of microbial biomass as well as methodological differences and contamination [24,25,54-56,60,61]. Unfavorable effect of microbiological and histopathological features on outcomes.

The strongest evidence identified for clinical outcomes was in cervical cancer research. High-grade cervical pathology and invasive disease were associated with persistent high-risk HPV infections, particularly HPV-16 and HPV-18 [8,31,32]. Cervicovaginal dysbiosis increased the likelihood of HPV persistence and lesion development, although this association is likely not causative [13,15,17,39,44].

Poor differentiation, deep stromal invasion, myometrial invasion, lymphovascular space invasion, tumour necrosis, high mitotic activity, and marked nuclear atypia were associated with aggressive disease behaviour in gynecologic cancers [1,2,12,18-20,57-59]. All of these features are still significant for prognosis, staging support, treatment planning, and follow-up [1,2,12,18-20,57-59].

Discussion

The goal of this comprehensive study was to synthesize the current evidence on the histopathological and microbiological features of gynecologic malignancies and premalignant diseases. The most convincing and consistent evidence was the presence of HPV-related LGT and cervical neoplasia. Across the included studies, HPV-16 and HPV-18 were the two most important carcinogenic HPV types, particularly in the context of cervical malignancies and CIN. These findings confirm the well-established role of persistent high-risk HPV infection in cervical carcinogenesis and its association with progressive epithelial dysplasia, HSIL, and INV [8,31,32].

The histopathology of cervical cancers was matched to the known sequence of HPV-induced changes. SCC continued to be the predominant histological type, and ADC was an important type with different HPV associations based on different histological types. Classifications and criteria as per the International Endocervical Adenocarcinoma Criteria and Classification (IECC) have led to a better classification of types of endocervical adenoma into HPV-dependent and HPV-independent endocervical ADCs, as these two types may have differences in morphology, pathogenesis, and prognosis, which is clinically relevant [33,35,36]. Based on these findings, cervical and LGT carcinogenesis should not be considered a microbiological or a histopathological process; these are all important, and their combined assessment, together with HPV status, should be applied to reach a more comprehensive understanding of disease.

Cervicovaginal dysbiosis has been identified as a key co-factor in HPV persistence and progression of lesions. Low *Lactobacillus* prevalence, high bacterial diversity, and anaerobic bacteria overgrowth were shown to be associated with high-risk HPV infection, with CIN and with cervical cancer in various studies [13,15,17,37-51]. These findings indicate that dysbiosis can foster a local microenvironment that involves chronic inflammation, an altered epithelial barrier function that can influence immune signaling, and impaired clearance of viruses. However, the majority of microbiome studies were cross-sectional or observational, and dysbiosis was considered an associated cofactor, but not necessarily a standalone cause of malignancy.

The importance of histopathological and molecular identification in endometrial cancer overtook microbiological evidence. Improved comprehension of endometrial carcinoma subtypes, prognosis, and clinical stratification is gained through traditional pathogenetic models and approaches of new molecular classification [18-20]. Potential differences in uterus or uterine tract microorganisms have been found amongst individual women with endometrial cancer in emerging microbiome studies [24,54-56]. These findings are, however, preliminary, as contamination during sampling, DNA extraction, sequencing, and/or bioinformatics may have a significant impact on upper genital tract and tumour material, which are low-biomass environments.

Much of the evidence for OC centers on both molecular and histopathological aspects of cancer, with a lack of definite evidence of any microbial cause of disease. HGSC is the primary OC histological type and is best defined through molecular pathways, genomic instability, and FT origin [21-23,57-59]. Few groups have conducted tumour microbiome studies, indicating the presence of bacterial DNA or tumour-associated microbial signatures in OC tissues [25,60,61]. To date, however, it remains unclear whether bacterial DNA detected in ovarian tumour tissue has biological significance or whether these microbial signatures play a role in carcinogenesis or tumour progression, or instead represent contamination, secondary colonization, or immune modulation.

Both an HPV-associated and HPV-independent pathway were identified among vulvar and vaginal cancers. There existed similarities between HPV associated vulvar and vaginal squamous neoplasia and cervical squamous carcinogenesis, particularly regarding HPV-16 predominance. But, HPV independent pathways of vulvar SCC exist as well, sometimes accompanied by differentiated VIN and chronic epithelial diseases [62-66]. This distinction is relevant as an HPV-associated lesion and an HPV-independent lesion might exhibit different precursor morphology, molecular changes, patient characteristics, and clinical presentation.

Limitations of Evidence Included in Review

Quality of evidence was noted to have several limitations. First, studies included were heterogeneous in design, population, sample size, anatomical sampling site, microorganisms, sequencing platform, histopathology, and outcome reporting. It was this heterogeneity that made direct comparisons between studies difficult and led to the absence of a quantitative meta-analysis.

Secondly, a lot of microbiome studies were cross-sectional, meaning that they could not draw conclusions about causation. While a high anaerobe bacterial community or a reduced dominance of *Lactobacillus* were often correlated with HPV persistence and severity of cervical lesions, these studies were unable to determine whether dysbiosis was an initial event leading to progression of lesions, or a result of epithelial and immune disease-related changes.

Third, samples analyzed from endometrial and ovarian tumours were samples with low biomass content. These studies are especially susceptible to contamination at the time of sampling as well as in steps of DNA extraction, sequencing, treating reagents, bioinformatics processing, etc. Microbial signatures observed in endometrial and OC should therefore be interpreted with caution, as they have yet to be confirmed by larger, well-controlled and longitudinal studies.

Fourth, HPV related studies included were generally stronger, as the HPV test has more standardization, HPV genotyping, and diaphragmatic confirmation of tumour is very well standardized. However, microbiome studies differed in type of sample collected, DNA extraction method, region of DNA sequenced, platform used for sequencing, whether contamination was checked or not, and bioinformatics analysis. This variation may affect microbial profiles and reproducibility.

Fifth, there was variable outcome reporting. Some studies placed an importance on analysis of HPV genotype distribution, others on analysis of lesion grade, histological subtype, immune markers, microbial diversity, or tumour‐associated bacterial DNA. This heterogeneity prevented production of pooled estimates and/or comparisons of effect sizes between studies.

Limitations of Review Process

This review has several methodological limitations. First, it was not prospectively registered in the International Prospective Register of Systematic Reviews (PROSPERO), and no formal protocol was publicly deposited before the review was conducted. Therefore, the possibility of protocol deviations or selective reporting cannot be completely excluded.

Second, despite searching multiple databases using a structured search strategy, the possibility of missing relevant studies due to differences in terminology, indexing, language, and database coverage cannot be excluded. In particular, search terms such as "microbiome," "dysbiosis," "tumour microbiota," and "gynecologic cancer" are evolving, and some relevant studies may not have explicitly used these terms.

Third, only those studies with extractable microbiological, histopathological, molecular microbiology, microbiome, or clinicopathological data were included. Studies reporting incomplete results were excluded, and result details were not provided in sufficient detail in some studies, potentially leading to selection bias.

Finally, only published data were used for review. What was not published in the article or part of the data sources was not retrieved from study authors. Accordingly, synthesis cannot be complete due to limited information or quality of information available in published reports.

Fifth, there may have been some publication bias and selective outcome reporting, which cannot be completely excluded. Although a meta-analysis was not done, funnel-plot analysis was not formally performed because of different designs, outcomes, and reporting structures of included studies.

Implications for Practice, Policy, and Future Research

The results confirm the value of HPV testing, HPV vaccination, screening, and pathological confirmation in the detection and prevention of cervical cancer [5-9,31,32]. Infection with high-risk HPV, particularly HPV-16 and HPV-18, continues to be the best-defined microbiological cause of gynecologic cancers [5-9,31,32]. Incorporating HPV testing into the context of forms of testing based on cytology and histopathology, along with molecular characterization of infection status and other markers, can enhance risk stratification and clinical decisions in the setting of cervical and LGT cancer [1,2,8,12,31-36].

Histopathology continues to be a diagnostic cornerstone in the workup of gynecologic cancer for clinical practice [1,2,12]. Subtype, grade, invasion, lymphovascular space invasion, necrosis, mitotic activity, precursor lesion type, and molecular classification are still important for prognosis and treatment planning [1,2,12,18-20,57-59]. Presence of a microorganism should not be regarded as a specific feature but should be judged within the context of pathological diagnosis [1,2,12,24,25,54-56,60,61].

Policy-wise, results further highlight the need for organized cervical cancer screening, HPV vaccination initiatives, HPV testing aligned with appropriate quality assurance measures, and standardized reports for pathology results [5-9,31,32]. Increased efforts can lead to less impact of HPV-related cervical, vulvar, and vaginal cancers [31,32,62-66].

Cervicovaginal microbiome profiling could also prove to be an efficient tool for identifying women at high risk of HPV persistence or progression of cervical lesions in the future, though it is too early to apply routinely in the clinic [13,15,17,37-51]. In future studies, contamination controls, uniformity of sequencing methods, standardized sampling protocols, clinically meaningful endpoints, and longitudinal design studies should be used [13,15,17,24,25,37-51,54-56,60,61].

The use of tumour microbiome findings in endometrial and OCs should, for now, be viewed only as exploratory and hypothesis-generating [24,25,54-56,60,61]. Future studies should also include microbiological profiling, histopathology, molecular classification, immune markers, tumour microenvironment (TME) analysis, and clinical outcomes [1,2,12,18-20,24,25,54-61]. Before microbiome-based biomarkers can be translated into clinical practice, larger multicentre studies with appropriate contamination controls are needed [24,25,54-56,60,61].

## Conclusions

High-risk HPV, especially HPV-16 and HPV-18, remains the most clearly established microbiological factor involved in gynecologic carcinogenesis, particularly in cervical cancer and LGT squamous neoplasia. Histopathology continues to play a central role in diagnosis, as it provides crucial details about tumour type, grade, invasion, precursor lesions, and features suggestive of aggressive disease. Cervicovaginal dysbiosis may contribute to HPV persistence and the progression of cervical lesions. However, current evidence suggests that it acts more as a supporting cofactor rather than as an independent cause of malignancy. In endometrial and OCs, the role of microbes is still not well established and remains largely exploratory, mainly because of differences in study methods and the risk of contamination in low-biomass tumour samples.

A combined approach that includes microbiology, histopathology, molecular classification, and immune profiling may help improve our understanding of gynecologic cancers in the future. Nevertheless, standardized research methods and long-term studies are still needed before microbiome-based markers can be confidently used in routine clinical practice.
